# Calcifications of the Knee’s Medial Compartment: A Case Report and Literature Review on the Adductor Magnus Tendon as an Uncommon Location and the Role of Ultrasound-Guided Lavage

**DOI:** 10.3390/diagnostics15050534

**Published:** 2025-02-22

**Authors:** Elena Jiménez-Herranz, Joao Vitor de Castro Fernandes, Juan José Ramos-Álvarez, Federico Del-Castillo-Díez, André Pedrinelli, Sofia Alvariza-Ciancio, Cristian Solís-Mencía, Federico Del-Castillo-González

**Affiliations:** 1 School of Sport Medicine, Radiology, Rehabilitation and Physiotherapy, Complutense University of Madrid, Pl. de Ramón y Cajal, 3, Moncloa-Aravaca, 28040 Madrid, Spain; 2 Hospital La Paz Institute for Health Research, P. º de la Castellana, 261, Fuencarral-El Pardo, 28046 Madrid, Spain; 3 Sports Medicine Departament, Hospital das Clínicas da Faculdade de Medicina da Universidade de São Paulo, R. Dr. Ovídio Pires de Campos, 333, Cerqueira César 05402-000, Sao Paulo, Brazil; 4 Department of Medicine, Faculty of Health Sciences, University of Deusto, Unibertsitate Etorb., Deusto, 48007 Bilbao, Spain; 5 Hospital Virgen de la Paloma, C. de la Loma, 1, Chamberí, 28003 Madrid, Spain

**Keywords:** calcific tendinopathy, magnus adductor, percutaneous lavage ultrasound

## Abstract

**Background:** This paper examines the diverse etiologies of medial knee pain, emphasizing the prevalence of calcification-related pathologies, such as Pellegrini–Stieda Syndrome (PSS), particularly in the medial collateral ligament (MCL) and adjacent structures. Furthermore, we present a case of calcification of the distal adductor magnus tendon (DAMT) insertion into the femoral condyle of the knee and describe its treatment using ultrasound-guided percutaneous lavage (UGPL). A narrative review was conducted based on a single case; it underscores the importance of accurate diagnosis using magnetic resonance imaging (MRI) to differentiate between various calcific conditions, guiding appropriate treatment strategies. **Case Presentation:** A 70-year-old patient presenting with severe medial knee pain, with a duration of 4 days, and functional impotence underwent X-ray, ultrasound, and magnetic resonance imaging (MRI) examinations, revealing calcification in the DAMT. Treatment consisted of UGPL. The patient’s pain level was assessed using the visual analog scale (VAS) initially and after 30 days of treatment. Upon initial assessment, the patient reported a VAS score of 9 out of 10. After 30 days of completing the treatment, the symptoms ceased. Follow-up imaging (X-ray, ultrasound, and MRI) showed only very tiny fragments of calcification remaining. **Conclusions:** UGPL is an effective technique for treating calcific tendinopathy of the DAMT insertion into the medial femoral condyle of the knee, offering significant pain relief and functional improvement. This case highlights the importance of considering this rare condition in the differential diagnosis of medial knee pain.

## 1. Introduction

Medial knee pain can arise from a variety of pathologies, including osteoarthritis, medial femoral condyle avulsion fractures, medial meniscopathy, medial patellofemoral ligament injuries, medial collateral ligament (MCL) injuries, and tendinopathies of the pes anserine and medial gastrocnemius tendons [1,2,3,4].

Some of these lesions are susceptible to developing hydroxyapatite deposition disease [5]. This is observed more frequently in the MCL [6] and less commonly in the bursa of the MCL [4] and the ischiochondylar portion of the adductor magnus [7]. Ossification of the MCL, whether due to direct injury or indirect mechanisms like microtrauma, results in pain and decreased range of motion. The grouping of all these pathological conditions, including the avulsion of the medial epicondyle [8], is classically known as Pellegrini–Stieda Syndrome (PSS) [8,9,10,11].

There is no unified consensus in the scientific literature regarding the precise anatomical location and mechanism of calcifications or ossifications around the medial femoral condyle. PSS encompasses a heterogeneous group of pathologies characterized by the presence of such calcifications or ossifications in the medial compartment of the knee. These can affect various structures, including the MCL origin, the area medial to the gastrocnemius medial head muscle origin, the adductor magnus tendon, and the posterior attachment of the medial patellofemoral ligament [6,7,12].

**Figure 1:**
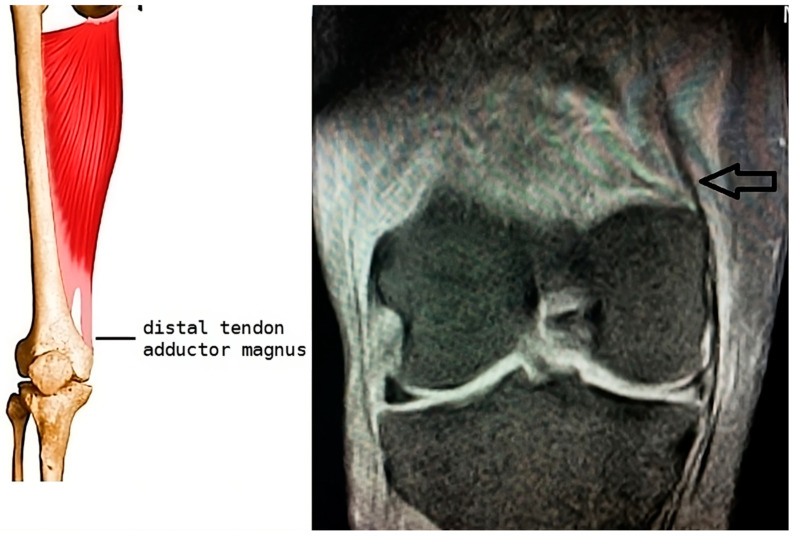
Diagram of a coronal section of the knee showing the area of insertion of the magnus adductor tendon in the internal femoral condyle and corresponding image in MRI (arrow).

Some authors distinguish the fibers inserting on the upper aspect of the tubercle as belonging to the long tendon of the adductor magnus, differentiating them from the insertions of the medial gastrocnemius and the medial collateral ligament of the knee [6,7]. Despite this, the close proximity of the MCL fibers and the ischiochondylar portion of the adductor magnus can complicate the differential diagnosis. Magnetic resonance imaging (MRI) plays a crucial role in specifying the location and type of calcifications, which facilitates a differential diagnosis [13] (Figure 1).

Identifying the location and type of calcification can be crucial for establishing a differential diagnosis from other causes of ossification or calcification in the region. These other causes might be responsive to different treatments previously described in the literature, such as conservative management, ultrasound-guided percutaneous lavage, open surgery, and ultrasonic percutaneous debridement [2,10,11,14,15,16]. Selecting the appropriate technique for each case can minimize the risk of treatment failure.

Recent studies have highlighted the importance of advanced diagnostic imaging and the role of minimally invasive treatments for managing calcific tendinopathies around the medial knee [2,14,17]. It is important to emphasize the need for a detailed understanding of the anatomical locations where ossifications typically occur, such as the MCL origin and the adductor magnus tendon, and suggests that ultrasound-guided percutaneous lavage (UGPL) may be an effective option for treating these rare calcifications [17]. By accurately identifying and targeting the source of calcification, physicians can select the most appropriate treatment plan, minimizing the risk of treatment failure and optimizing patient outcomes. The growing evidence for the use of minimally invasive procedures, such as UGPL, is shaping the future of management for conditions like Pellegrini–Stieda Syndrome and other similar pathologies in the knee [14,15,16].

This case presents a rare instance of adductor magnus tendon calcification (AMTC) treated with ultrasound-guided percutaneous lavage (UGPL). The diagnostic complexity—stemming from the overlapping anatomical structures and rare site of mineralization—and the use of UGPL as a minimally invasive treatment underscore the clinical significance of this report. To our knowledge, this is among the few documented cases of AMTC managed with UGPL, highlighting its potential as a safe and effective therapeutic approach.

By identifying and treating an uncommon site of calcific tendinitis, this report aims to contribute to the understanding of rare medial knee pathologies and their management. It further emphasizes the role of advanced imaging and minimally invasive interventions in improving diagnostic accuracy and patient outcomes.

## 2. Case Description

### 2.1. Patient Information

A 70-year-old man presented with acute right knee pain, difficulty walking, and a reduced range of motion (ROM), accompanied by mild inflammation on the medial aspect of the joint. The symptoms began one week earlier, following an episode of overexertion. Notably, the patient reported no history of trauma or prior treatment for the knee. His medical history was unremarkable, with no evidence of crystalline arthropathy, inflammatory arthropathy, or hypercalcemia. During the initial physical examination, the patient rated his pain as 9 out of 10 on the visual analog scale (VAS) [18]. The absence of trauma or other systemic conditions is important to highlight in this context, emphasizing the need for a differential diagnosis focused on intrinsic joint pathology.

### 2.2. Clinical and Imaging Exams

**Figure 2:**
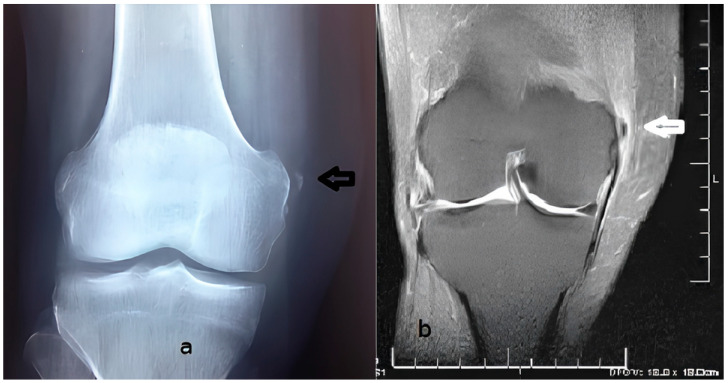
(**a**) Anteroposterior radiograph of the right knee showing calcification adjacent to the internal femoral condyle (black arrow). (**b**) Coronal PD-FS image of the right knee showing a hypointense calcification adjacent to the internal femoral condyle (white arrow).

**Figure 3:**
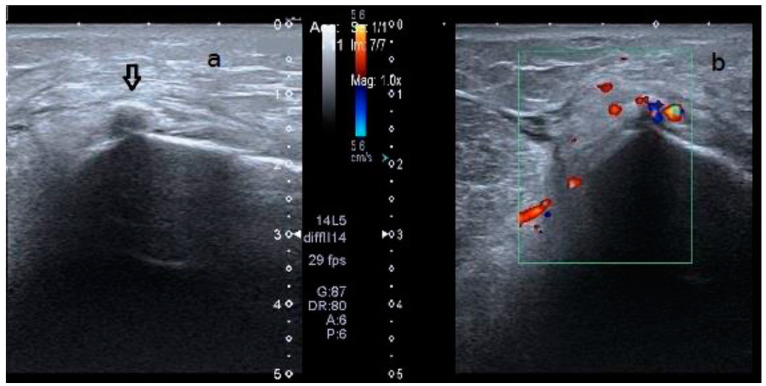
(**a**) Long-axis sonogram showing an echogenic focus with posterior acoustic shadow overlying or adjacent to the internal femoral condyle (black arrow). Black Arrow = needle. (**b**) Hypervascularization is observed in the color Doppler scan.

The physical examination revealed a painful and swollen area on the medial side of the knee, with pain worsening upon bending the knee or applying pressure to the joint. X-ray, ultrasound, and MRI examinations (Figure 2 and Figure 3) identified a curvilinear calcification near the medial femoral condyle, corresponding to the symptomatic area. Ultrasound revealed a curved echogenic line, 1 cm long, with an acoustic shadow on the internal femoral condyle (Figure 3). MRI demonstrated a linear, hypointense feature in all sequences, situated along the course and insertion of the distal tendon of the adductor magnus (Figure 2). These findings confirmed the diagnosis of adductor magnus tendon calcification (AMTC).

Considering the techniques described for treating calcifications of the medial aspect of the knee, UGPL was chosen as the treatment modality. This decision was made based on the excellent results UGPL has demonstrated in managing calcific tendinopathy of the shoulder [16,19].

### 2.3. Technique Description

**Figure 4:**
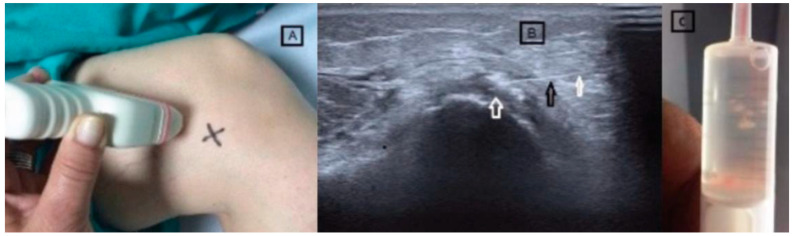
(**A**) Photograph showing patient and ultrasound transducer positioning for sonographically guided percutaneous lavage of the calcified tendinopathy, “X” denotes the needle skin entry site. (**B**) Long-axis sonogram showing the needle located over the calcification (black and white arrow). (**C**) Syringe with calcium material obtained in the lavage.

After receiving a thorough explanation of the procedure, the patient provided written informed consent and authorization for the anonymous publication of their case. The procedure was performed with the patient positioned on their right side. Under continuous ultrasound guidance, the calcification near the medial femoral condyle was identified, and the skin was marked to establish the needle insertion point (Figure 4). Ultrasonography assessments were also used to evaluate potential knee conditions such as osteoarthritis (KOA), effusion, synovitis, bursitis, and Baker’s cyst. These evaluations confirmed no signs of effusion, synovitis, or associated bursitis.

The area was cleaned with povidone-iodine, and strict aseptic technique was maintained throughout the procedure. To ensure sterility during ultrasound-guided aspiration, the ultrasound probe was disinfected before the procedure with a disinfectant solution as recommended by the device guide. Additionally, a sterile, single-use probe cover was applied, and sterile coupling gel was used.

Equipment preparation included a 5-mL syringe with 2% mepivacaine for local anesthesia, several 10-mL syringes filled with physiological saline for lavage, and a 5-mL syringe containing 1 mL of triamcinolone (40 mg). After administering local anesthesia at the puncture site, an 18G needle was inserted under ultrasound guidance.

With continuous ultrasound visualization, pressure impulses were applied to the syringe plunger and then released to facilitate breakdown of the calcification. These pulses were repeated until the calcified material fragmented, visible as particulate matter in the syringe. Once the syringe was full, it was replaced with another containing saline solution, while keeping the needle in place. The lavage process was repeated until no further calcified material could be withdrawn. The fragmented calcified material was collected in three syringes for histological analysis, which confirmed the presence of hydroxyapatite crystals.

To complete the procedure, 1 mL of triamcinolone was injected through the needle before its removal. The entry point was covered with sterile gauze. The patient tolerated the procedure well, with no adverse reactions noted during or after the intervention. Post-procedure recommendations included resuming normal activities while avoiding prolonged standing during the week following treatment.

**Figure 5:**
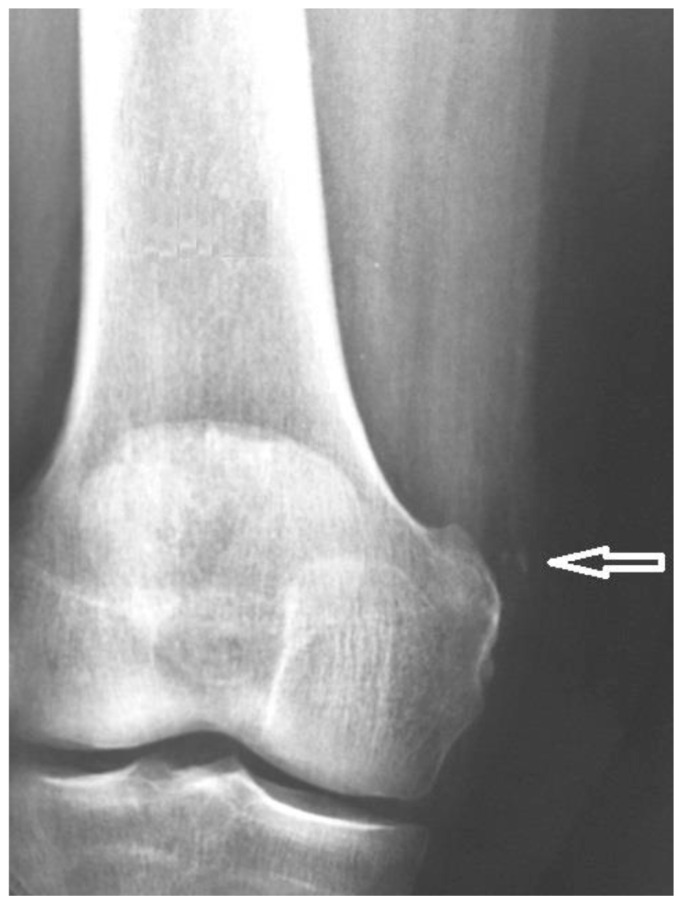
Anteroposterior radiograph at 1-month post-UGPL. Only small residual fragments of the calcification are visible (arrow). The patient was asymptomatic (EVA 0).

At the one-month follow-up, the patient reported complete resolution of pain and inflammation. X-ray and ultrasound examinations revealed nearly complete disappearance of the calcification, with only minor fragments remaining (Figure 5). The patient’s pain score improved to 0/10 on the visual analog scale (VAS) [18].

**Figure 6:**
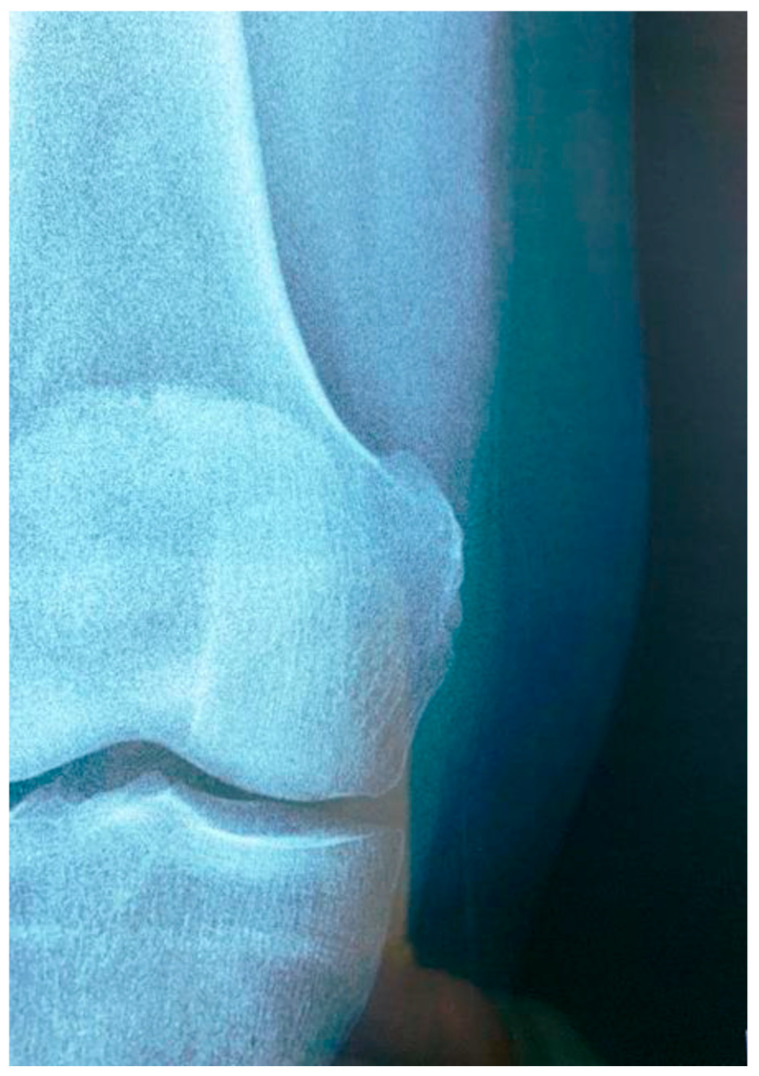
Anteroposterior plain radiograph at 6 months post-UGPL. No calcifications are visible. Patient was asymptomatic (EVA 0).

At the six-month follow-up, the patient remained asymptomatic (VAS: 0), with imaging revealing no residual calcification (Figure 6).

## 3. Discussion

The shoulder is the most common location for calcific tendinopathy. However, calcifications can occur less frequently at other sites, including the hamstring tendon insertions on the ischial tuberosity, the proximal or distal deltoid insertions, the gluteus maximus, the popliteus, and within the wrist, elbow, knee, ankle, and foot [2,20,21,22,23,24,25]. AMTC is an infrequent finding. To our knowledge, this is the first reported case of successful treatment using UGPL for this condition.

Calcifications on the medial aspect of the knee are most commonly associated with Pellegrini–Stieda Syndrome (PSS), primarily considered a post-traumatic pathology, which can also occur in pediatric patients [6,7,11,12,26,27]. However, not all periarticular calcifications affecting tendons, ligaments, and occasionally the bursae of the knee are associated with a history of trauma [27,28]. Therefore, a differential diagnosis is important to determine the most appropriate treatment strategy, as this anatomical region can also be affected by ossification such as avulsion fractures, myositis ossificans, and heterotopic ossification [29], which differs from calcification. Some conditions that can manifest as calcifications in this area include those secondary to metabolic disorders such as gout, chondrocalcinosis (calcium pyrophosphate deposition disease), and tumors [29,30,31]. In this case, the patient presented with hydroxyapatite crystal deposition, the most common form of soft tissue calcification [5,32,33].

Some authors distinguish several stages in the formation of calcifications: a formative phase (Type I), a resting phase (Type II), and a resorption phase (Type III) [34,35,36]. In our case, both radiographic and ultrasound imaging findings were consistent with a calcification in the formative phase. The X-ray demonstrated a typical cloudy appearance with a well-defined oval shape [37] (Figure 2), while ultrasound revealed a dense calcification with significant posterior acoustic shadowing [5,37] (Figure 3). Following treatment, imaging revealed vague borders and a more transparent appearance, indicating a possible resorption phase (Type III) [5,35] (Figure 6). However, to establish a comprehensive understanding and differential diagnosis of calcifications within the internal compartment of the knee, MRI is crucial [5,6,7,35]. In this case, MRI played a pivotal role in delineating the lesion’s precise anatomical location. The patient had not experienced prior trauma, ruling out a medial epicondyle avulsion. The anatomical position of the calcification corresponded to the insertion of the adductor magnus, situated superior and lateral to the insertion of the medial collateral ligament and proximal to the gracilis muscle insertion on the tibial plateau (Figure 2). This finding facilitated the decision to employ ultrasound-guided percutaneous lavage (UGPL) as the treatment modality.

Various degenerative or reactive causes have been described in the etiopathogenesis of calcifications, with or without previous direct or indirect trauma. These mechanisms may lead to periosteal proliferation with direct metaplasia of periarticular structures onto a previously damaged tendon [38] or a reactive process followed by tendon resorption and remodeling [34]. In this case, the patient reported no history of trauma after a thorough examination, and the calcification was in the formative phase, causing significant pain and functional impairment. Therefore, we decided to initiate treatment rather than wait for the completion of the evolutionary phases described in the process of tendinous calcification [34,35,36].

Multiple treatment options are available for calcifications on the medial aspect of the knee, and their effectiveness may vary depending on the location and type of lesion. Classically, surgical intervention, involving excision of the ossified fragment and MCL repair, was indicated when conservative treatment failed [15,39]. Previously, only two studies have reported positive outcomes using minimally invasive methods for treating PSS lesions: ultrasound-guided percutaneous debridement [14] and UGPL [28]. Our group previously reported a case of MCL bursa calcification treated with UGPL [4], representing an uncommon location for calcifications in the medial knee compartment. This review presents another unusual calcification in this region, specifically within the adductor magnus tendon, adjacent but external to the MCL.

The favorable outcomes previously achieved with this technique in calcifications at other locations led us to apply it in this case, with excellent results. The therapeutic intervention yielded excellent radiological improvements and short-term and long-term clinical outcomes. These findings suggest the potential utility and efficacy of UGPL as a viable treatment option for calcific tendinopathy affecting the knee, reinforcing its role as a valuable therapeutic strategy in the management of such conditions. We believe that it is essential to differentiate between ossification and calcification in the diagnosis of PSS, regardless of location, to establish a specific and effective treatment plan.

Concerning this case, the visual analog scale (VAS) was employed to assess the patient’s pain levels in this case, providing a clear quantitative measure of symptom improvement post-procedure. The VAS is widely recognized as a reliable tool for evaluating pain intensity, with scores ranging from 0 (no pain) to 10 (worst possible pain) [18]. The patient’s VAS score of 0/10 at both the one-month and six-month follow-ups indicated complete resolution of pain and confirmed the effectiveness of the intervention. By measuring the pain reduction associated with the calcification removal procedure, it helps clinicians understand not only the physical resolution of the pathology but also the patient’s return to normal function. These assessments can complement imaging studies and provide a holistic view of treatment outcomes, particularly in musculoskeletal conditions where pain and function are tightly linked [40]. Functional assessments offer valuable insights into the impact of treatment on daily activities and quality of life. Future studies could benefit from integrating both objective imaging findings and subjective functional measures to provide more comprehensive evaluations of treatment efficacy.

The success of minimally invasive treatments for tendinous and ligamentous calcifications, as demonstrated in this case, suggests that these approaches could become a first-line treatment option, potentially preferred over conservative management. Given the rapid recovery, resolution of pain, and favorable imaging outcomes observed in this patient, these techniques could be a viable alternative to more invasive interventions. Surgical options might then be reserved for cases where minimally invasive methods fail, offering a more targeted and effective approach to managing calcific tendonitis.

### 3.1. Recommendations for Future Studies

To further advance the understanding and management of this condition, future research is warranted. Future studies should focus on longitudinal and functional assessments to evaluate the long-term efficacy and safety of treatments for calcific tendinopathy affecting the adductor magnus tendon insertion in the knee. Population-based studies can provide insights into the true incidence of this condition across different demographics, and comparative analyses should compare the effectiveness of ultrasound-guided percutaneous lavage with other treatment modalities.

### 3.2. Strengths and Limitations of the Study

The strengths of this study lie in its comprehensive evaluation of UGPL as a treatment modality for calcific tendinopathy of the adductor magnus insertion in the knee. The inclusion of longitudinal follow-up data allowed for a thorough assessment of treatment efficacy and durability over time. Additionally, the study’s multidisciplinary approach, involving collaboration between orthopedic surgeons, radiologists, and researchers, enhanced the robustness of the findings. However, limitations include the relatively small sample size and the lack of a control group for comparison, which may limit the generalizability of the results.

## 4. Conclusions

In summary, calcific tendinopathy affecting the insertion of the long tendon of the adductor magnus in the knee is a rare occurrence, possibly due to limited available data. However, it should be considered during the differential diagnosis of pain affecting the medial aspect of the knee joint. UGPL appears to be a promising treatment for this condition and may emerge as a first-line treatment option, although further studies are needed to validate its efficacy and determine the true incidence of adductor magnus calcification. This underscores the importance of individualized treatment approaches for the various injuries that may be associated with calcification on the inner side of the knee. Continued research in this area is essential for refining diagnostic and therapeutic strategies.

## Data Availability

All data generated or analyzed during this study are included in this published article. Further inquiries can be directed at the corresponding author upon reasonable request.
